# Clinical Equivalence of Clinifibre® and FiberWire® Ultra-High Molecular Weight Polyethylene Suture for Graft Preparation in Unilateral Anterior Cruciate Ligament Reconstruction: A Single-Blind Randomized Study

**DOI:** 10.7759/cureus.83947

**Published:** 2025-05-12

**Authors:** Taufiq Panjwani, Tanmay Datta, Chandan Kumar, Manish Maheshwari, Debdutta Chatterjee, Ashok K Moharana, Deepak TS

**Affiliations:** 1 Orthopedics and Traumatology, Shalby Hospital, Ahmedabad, IND; 2 Orthopedics, Institute of Post-Graduate Medical Education and Research and Seth Sukhlal Karnani Memorial Hospital (IPGM&amp;ER and SSKM Hospital), Kolkata, IND; 3 Orthopedics and Traumatology, Ganesh Shankar Vidyarthi Memorial (GSVM) Medical College, Kanpur, IND; 4 Orthopedics, Shalby Hospital, Indore, IND; 5 Orthopedics, Burdwan Medical College, Burdwan, IND; 6 Clinical Affairs, Healthium Medtech Limited, Bengaluru, IND

**Keywords:** anterior cruciate ligament, laxity, quality of life, surgical site infection, uhmwpe sutures, ultra-high molecular weight polyethylene (uhmwpe)

## Abstract

Introduction

Arthroscopic anterior cruciate ligament reconstruction (ACLR) is the gold standard for restoring rotational stability of the injured knee. The success of ACLR depends on surgical preparation and technique and is indicated by post-operative complications and functional outcomes. The study investigated the clinical equivalence of Clinifibre^®^ and FiberWire^® ^ultra-high molecular weight polyethylene (UHMWPE) sutures for graft preparation during unilateral primary ACLR.

Methods

Skeletally mature adults (18-50 years) undergoing unilateral primary ACLR with hamstring autograft were randomized to Clinifibre^®^ (n=36) and FiberWire^®^ (n=36) groups (March 2022 to September 2024). The primary endpoints, surgical site infection (SSI) and revision surgery due to ACL graft failure, were assessed, along with Lachman test, pivot shift test, hospital stay, single leg-hop test, scores of Lysholm knee, Tegner activity, International Knee Documentation Committee subjective knee evaluation form, pain, Knee Osteoarthritis and Outcomes Score quality of life (KOOS-QOL), intraoperative and post-operative complications, suture handling characteristics, return to normal activities, return to work, return to pre-injury sports, mobilization period with crutches, and adverse events. The level of significance was tested at p<0.05.

Results

The rate of SSI (at skin suturing site) was not significantly different between the groups and was found only in one subject in the FiberWire^®^ group. During the study, revision surgery was not required in either arm. Return to day-to-day activity, return to work, return to pre-injury sports, and mobilization with crutches and single leg-hop score were comparable. The ACLR has improved anteroposterior laxity, rotational stability, KOOS-QOL, pain, and knee-related scoring in both groups.

Conclusion

The primary and secondary outcomes confirmed clinical equivalence of Clinifibre^®^ and FiberWire^®^ sutures. An overall recovery concerning laxity, pain, knee stability, KOOS-QOL, and functional outcomes further affirmed the efficacy of UHMWPE sutures for graft preparation during primary ACLR.

## Introduction

The most common orthopedic injury, the anterior cruciate ligament (ACL) injury, hinders day-to-day activity and leads to chronic instability [1,2]. The annual incidence of ACL injury in the United States alone is 68.6 per 100,000 person-years [3]. The rate of ACL tears in India is as high as 86.5% [4]. Since the ligament preserves anteroposterior and rotational stability of the knees [5], ACL tears can often be a career-defining injury for young and active people [6]. Arthroscopic ACL reconstruction (ACLR) surgery is a gold standard that, together with rehabilitation protocols, can help in achieving the pre-injury activity level [2]. A large worldwide ACLR survey reported that India performs 46.0% ACLR annually [7]. The success of ACLR surgery is determined by the rate of post-operative complications. Controlling the risk factors by the use of antibiotic prophylaxis and the execution of proper surgical preparation and technique may elevate the rate of success [8].

Arthroscopic ACLR with suture augmentation technique can be performed in all age groups to protect the ligament during early rehabilitation [9]. Approximately 94% of surgeons in India use hamstring tendon autograft for ACLR [2]. Individuality is maintained while choosing hamstring graft length, graft preparation technique, and fixation method. An ideal graft preparation and elongation requires the quality of the tissue and stitching technique, as well as the suture strength and number of suture throws [10]. Ultra-high molecular weight polyethylene (UHMWPE) sutures with well-aligned microstructure polythene chains, excellent tensile strength, and load-bearing capacity are highly used in the construction of ACL and other ligaments and tendons [11]. The study aimed to evaluate the clinical equivalence of two UHMWPE braided sutures, Clinifibre® (Healthium Medtech Limited, Bengaluru, Karnataka, India) and FiberWire® (Arthrex, Naples, FL, USA), for graft preparation in patients undergoing arthroscopic unilateral primary ACLR. We hypothesized that graft preparation with UHMWPE sutures may contribute successfully to adequate healing and ligamentization of the tendon graft, enhancing quality of life (QOL), clinical and functional outcomes, and satisfaction in patients undergoing ACLR.

## Materials and methods

Study design

The prospective, two-arm, parallel group, single-blind, randomized study was conducted at five centers across India between March 2022 and September 2024. The primary objective was to investigate the incidence of residual risks of sutures (infection, suture breakage, allergy, and inflammatory reaction) within 52 weeks of surgery, along with the secondary objectives to evaluate the clinical outcomes, objective and patient-reported functional outcomes, patient-related QOL, intra- and post-operative complications, intraoperative handling of sutures, return to activity post-operatively, tissue reaction, material problems, and other adverse events (AEs) among the Clinifibre® and FiberWire® groups.

Ethical approval

The study was designed and conducted in compliance with the Principles of International Council for Harmonisation of Technical Requirements-Good Clinical Practice (ICH-GCP E6 R2) guidelines, European Standard (EN) International Organization for Standardization (ISO) 14155:2020 guidelines, MDR (EU) 2017/745, Indian Medical Devices Rules (MDR) 2017, and Indian New Drugs and CT rules. Clinical Trial Registry of India (CTRI) reviewed and approved the study (CTRI/2021/09/036704, Dated 21/09/2021). The study was reported in accordance with ICH-GCP E3 and EN ISO 14155:2020 guidelines.

Study participants

Skeletally mature adults (18-50 years) undergoing unilateral primary ACLR, requiring UHMWPE suture for hamstring autograft preparation, and having a contralateral healthy knee (on clinical examination) were included. Subjects with physical activity classified by the Tegner scale ≥1 were included in the study after signing written informed consent.

Subjects were excluded if their body mass index was <18.5 or >30 kg/m^2^ and glycated hemoglobin level was >10%, were pregnant, or had posterior cruciate ligament injury, other collateral ligament injury, generalized ligamentous laxity (Beighton score ≥4 or defined as the simultaneous presence of joint hypermobility at the four limbs and axial skeleton, with involvement of both the major and minor joints), osseous fractures or trauma that could impair rehabilitation, requiring surgical correction for obvious change in the alignment of the mechanical axis (varus or valgus malalignment >3º), or other indication-based exclusion. Subjects who were professional athletes, heavy smokers, undergoing ACLR using bone-patellar tendon-bone graft, having a history of bleeding or connective tissue disorder or congenital disease (predisposing patient for articular cartilage damage), allergic to polyethylene or similar products, or not achieving 90° of flexion in the affected knee before surgery were excluded. Additionally, subjects who were participating in other trials, had experimental drug/device in the prior 30 days of the study enrolment, could not comply with surgical procedure/rehabilitation protocol or complete the follow-up visits, employee of the investigator or study site, involved with the current study or any other study under same investigator or study site were also excluded.

Intervention

The study compared two established UHMWPE sutures, Clinifibre® and FiberWire®. Both are sterile synthetic multifilament braided non-absorbable (not degraded or weakened by the action of tissue enzymes) surgical sutures prepared from fibers of UHMWPE.

Study setting

The Department of Orthopedics of five tertiary care centers - Shalby Hospital (Indore, Madhya Pradesh, India), GSVM Medical College (Kanpur, Uttar Pradesh, India), Shalby Hospital (Ahmedabad, Gujarat, India), Institute of Post-Graduate Medical Education & Research and SSKM Hospital (Kolkata, West Bengal, India), and Burdwan Medical College (Burdwan, West Bengal, India) - were involved in this study. Ethical Committee of each site approved the study: Ethics Committee of Shalby Hospital Indore (EC-SHI) at site 1 (approval dated January 19, 2022); Ethics Committee of GSVM Medical College at site 2 (approval no. EC/50/March/2022, dated March 16, 2022), Ethics Committee of Shalby Limited at site 3 (approval dated January 17, 2022), Institutional Ethics Committee of IPGME&R Research Oversight Committee at site 4 (approval no. IPGME&R/IEC/2022/262, dated April 26, 2022), and Institutional Ethics Committee of Burdwan Medical College at site 5 (approval no. BMC/IEC/098, dated June 22, 2023).

Study procedure

The study participants underwent pre-operative evaluation to assess their clinical condition and comorbidities. All the surgeries were performed according to standard local institutional policy by the same specialized consultant knee surgeon with recognized expertise in ACLR. The desired graft was harvested from a separate incision and pre-tensioned on the suture board. The tendon graft was prepared and strengthened with either Clinifibre® or FiberWire® sutures, and the free ends were stitched. The pre-prepared tibial and femoral tunnels were introduced with the graft using a combination of direct vision and instrumentation. Once the graft was in situ and under tension, it was fixed on the femoral and tibial sides using the institutional standard procedure. All incisions were sutured and bandaged, as per local protocols. Local institutional protocol was followed for anesthesia, deep vein thrombosis prophylaxis, and antibiotic use. The standard ACL rehabilitation program was performed as per the institutional protocol. All other care was routine, including postoperative care.

The study included one screening visit, which took place between 12 weeks and 1 day before surgery, and six study visits were conducted on day 0 (baseline visit/visit 1/day of surgery), day of discharge ([DOD] visit 2), week 1-3 (±day 3, visit 3), week 6-12 (±day 7, visit 4), week 26 (±day 7, visit 5), and week 52 (±day 7, visit 6).

Demographics and other relevant characteristics

Subject’s ethnicity, age, gender, weight, body mass index, and history of alcoholism and smoking were recorded at screening. Pulse rate, respiratory rate, temperature, and systolic and diastolic blood pressure were measured, along with a recording of any medical/surgical history. Additionally, systemic examinations were conducted, including an assessment of general appearance, edema, and lymph nodes for any abnormalities. Details related to the ACL injury were also noted.

Study outcomes

Primary Endpoints

The primary endpoint of the study was to assess the proportion of subjects having surgical site infection (SSI) and revision surgery due to ACL graft failure on DOD, week 1-3, week 6-12, and week 52. SSI is defined as superficial SSI, occurring within 30 days after the surgery, and deep or organ/space SSI, occurring up to 90 days after the surgery. In case of revision surgery, the proportion of subjects with bacterial growth on suture culture (if suture removed), suture breakage, inflammatory reaction or suture granuloma (confirmed by histopathology), and any allergic reactions post-surgery not attributable to any other medications or drugs (confirmed by histopathology) were recorded.

Secondary Endpoints

Clinical outcomes using the Lachman test, Pivot shift test, and length of hospital stay were measured. The number of days that subjects spent in the hospital was recorded on DOD. Anteroposterior laxity by the Lachman test was tested by a single surgeon after stabilizing the femur and applying an anterior force on the tibia of the subject (lying supine with knee flexed around 30°), without restraining axial rotation. The result of the injured knee was presented as grade 0, 1, 2, and 3, indicating the translation amount of <3 mm (proprioceptive appreciation of a positive test), 3-5 mm (visible anterior translation of the tibia), 6-10 mm (passive subluxation of the tibia), and >10 mm (active subluxation of the proximal tibia), respectively [12]. Pivot shift test for checking the rotational stability of the subject was performed based on the principle of applying valgus torque and internal rotation to the leg, when the knee is fully extended and then gently flexed to around 40°. A grading system was used: grade 0 (normal), grade 1 (glide), grade 2 (clunk), and grade 3 (locked subluxation) for assessing the pathologic motion [13]. The objective functional outcome of the subjects was assessed via the single leg-hop test, which was performed by standing on the leg to be tested and then hopping and landing on the same limb [14]. The distance hopped at the level of the great toe was measured and recorded at week 52.

Patient-reported functional outcomes of the Lysholm knee scoring scale, Tegner activity score, International Knee Documentation Committee (IKDC) subjective knee evaluation form, and pain score on visual analogue scale (VAS) were recorded. The Lysholm scale consists of eight subscales: pain (25 points), instability (25 points), locking (15 points), swelling (10 points), limp (5 points), stair climbing (10 points), squatting (5 points), and need for support (5 points), assigned an arbitrary score on an increasing scale, where higher scores indicate a better outcome [15]. The Tegner activity scale is a one-item score validated scale for evaluating activity level related to knee stability. On this scale, 0 represents disability due to knee problems and 10 represents competitive sports like national or international level soccer [15]. The IKDC consists of 18 items regarding symptoms (7 items), sports activities (10 items), and function (1 item), scored on a 0 (total limitation) to 100 (no limitations) Likert scale. A transformed total score was used to interpret higher levels of function and lower levels of symptoms [16]. Pain in the operated knee was reported by the subject using the 100-mm VAS: 0-4, no pain; 5-44, mild pain; 45-74, moderate pain; and 75-100, severe/worst pain.

Intraoperative profile of the subjects, including length of surgery (time from skin incision to the end of skin closure), antibiotic prophylaxis, anesthesia, length and diameter of ACL graft, source of graft, type of graft, graft preparation time, implant used for femoral and tibial fixation, thrombosis prophylaxis, blood transfusions, meniscal repair, and outcome of surgery, was recorded. Overall intraoperative handling of study sutures for graft preparation was rated on day 0 on a five-point scale: 1, poor; 2, fair; 3, good; 4, very good; and 5, excellent. Additionally, intraoperative complications and suture-related challenges were recorded. Post-operative complications, viz. graft failure, unexpected poor range of movement (stiffness), excess bleeding, and continued swelling, were also noted.

Knee-related QOL was measured using the Knee Osteoarthritis and Outcomes Score (KOOS). The four items of KOOS-QOL were scored as 0-4 on a Likert scale, with a higher score indicating better QOL [17]. Post-operative time to return to normal activities, work, and pre-injury sports, and mobilization period with crutches after the surgery were recorded. Tissue reaction, material problems, and other AEs were also investigated.

Sample size

A delay in revascularization of the graft, due to excessive tension, inadequate post-operative immobilization, infection, or immune reactions, postpones graft integration and results in graft failure [18]. Therefore, the proportion of subjects with ACL graft failure, as reported by a previous study [19], was considered for sample size calculation in the FiberWire® arm. The anticipated proportion of subjects with the ACL graft failure in the FiberWire® suture arm was assumed as 3.5%, i.e., π1=0.035. Assuming a type I error of 5%, power of 80%, and a difference of 1.4% for the proportion of patients having ACL graft failure in the Clinifibre® arm (π2=4.9%, 0.049), the sample size requirement was calculated as 28 in each arm (with 15% margin of non-inferiority). Considering the post-randomization withdrawal and exclusion of 25%, the sample size was increased to 72 (n=36 in each arm). Competitive enrolment was followed at five centers to meet the prescribed enrolment of 72 subjects within the assigned time. Any center after recruiting up to 18 subjects could further recruit up to 36 subjects, until the prescribed enrolment of 72 subjects was completed across all five centers. Overall, a sample size of up to 36 subjects was allowed for each center.

Randomization and blinding

The 72 subjects were randomized using block randomization in a 1:1 ratio, stratified per trial site, to receive either of the two sutures (Clinifibre® or FiberWire®). An independent programmer has generated a computer-based, automated randomization number before recruiting an eligible subject to the study. The randomization codes were issued to the sites in sequentially numbered, opaque, sealed envelopes (SNOSE). As the study was single-blind, the study intervention was only blinded to the subjects.

Statistical analysis

The statistical software SPSS Version 28.0 (IBM Corp., Armonk, NY, USA) was used to analyze primary and secondary outcomes of the subjects having complete data on the primary effectiveness parameter till the end of the study, without any major protocol deviations (i.e., intent-to-treat or full analysis set). The normality of all variables was tested using the Shapiro-Wilk test. The quantitative variables were expressed as mean±SD (t-test/Mann-Whitney U test was used depending on data distribution) and qualitative variables as proportions/percentages (chi-square test was used). The primary endpoint, proportion of subjects with residual risks of sutures within 52 weeks of the ACLR, was compared using the chi-square test. Normally distributed continuous data of analgesics, onset of pain, return to pre-injury sport, and total scores of Lysholm and IKDC were analyzed using the t-test. The Mann-Whitney U test was used to calculate the distribution-free data of time lapse from injury to ACLR, length and diameter of ACL graft, graft preparation time, number of sutures used for graft preparation, length of surgery, post-operative pain, duration of hospital stay, time to walk without crutches, return to day-to-day activity/work, number of stairs climbed, Tegner activity level scale, and length of single leg-hop. The level of significance was tested at p<0.05.

## Results

**Figure 1:**
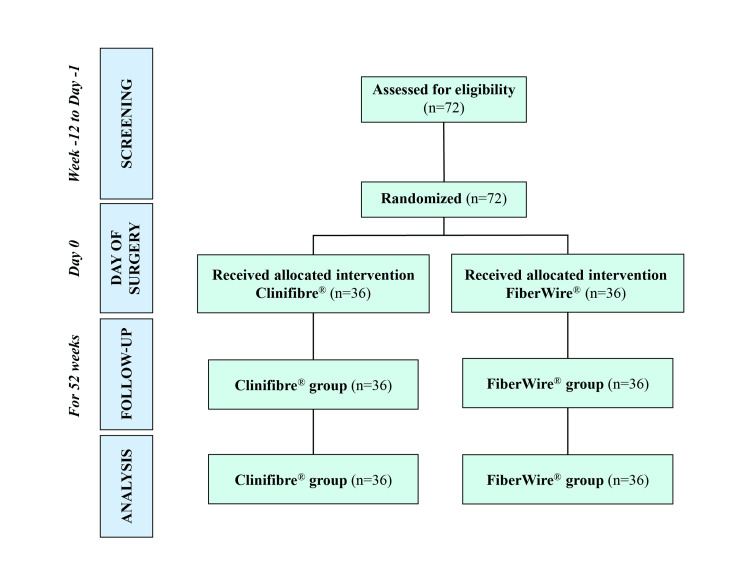
Consort flowchart

A total of 72 participants were screened between March 2022 and September 2023 and randomized to Clinifibre® (n=36) and FiberWire® (n=36) groups (Figure 1).

Demographics and other relevant characteristics

Among 72 study participants, 94.4% and 100.0% of subjects in the Clinifibre® and FiberWire® groups, respectively, were Indians; the rest in the Clinifibre® group were non-Indian Asians (p=0.10). Physical examination revealed abnormal joints and extremities in both groups (p=1.00). Prevalence of ACL injury was higher in male counterparts of both the Clinifibre® (66.7%) and FiberWire® (83.3%) groups (p=0.15). Proximal ACL tear was noted in the majority of subjects in the Clinifibre® (63.9%) and FiberWire® (50.0%) groups, followed by central 1/3 (30.6% vs. 47.2%) and distal tear (5.6% vs. 2.8%) (p=0.33). The reason for ACL tear in both Clinifibre® and FiberWire® groups was mostly injuries from falling (83.3% vs. 66.7%); accident and sports injury were the other reasons (p=0.40). Other characteristics of the participants are presented in Table 1.

**Table 1:** Characteristics of subjects undergoing anterior cruciate ligament reconstruction Data are presented as mean±standard deviation or frequency (percentage). n is the number of patients. MWU, Mann-Whitney U test

Subject characteristics	Clinifibre^®^ (n=36)	FiberWire^®^ (n=36)	Statistical test	P-value
Age (years)	30.1±8.1	31.4±8.8	t-test	0.965
Body mass index (kg/m^2^)	24.8±2.9	25.0±2.9	t-test	0.971
Pulse rate (beats/minute)	79.2±8.1	81.6±5.8	t-test	0.159
Respiratory rate (breaths/minute)	18.3±1.3	17.9±1.7	t-test	0.770
Temperature (ºF)	97.9±0.6	97.9±0.5	t-test	0.588
Systolic blood pressure (mmHg)	119.7±8.3	122.7±7.9	t-test	0.327
Diastolic blood pressure (mmHg)	77.5±6.2	79.7±6.7	t-test	0.990
Alcohol consumption	2 (5.6)	5 (13.9)	Chi-square	0.233
Smoking	1 (2.8)	3 (8.3)	Chi-square	0.303
Medical/surgical history	3 (8.3)	4 (11.1)	Chi-square	0.691
Time lapse from injury to surgery (days)	191.3±293.4	185.2±205.8	MWU	0.602
Operative knee				
Right knee	19 (52.8)	16 (44.4)	Chi-square	0.479
Left knee	17 (47.2)	20 (55.6)		

Primary outcomes

SSI at the site of skin suturing was noted in only one (2.8%) subject in the FiberWire® group at week 6-12 (p=0.31) but none in the Clinifibre® group. In addition, revision surgery was not required to be performed during the course of the study in subjects in both the Clinifibre® and FiberWire® groups.

Secondary outcomes

During screening, associated meniscus injury was identified in 58.3% (19.4% lateral tear and 38.9% medial tear) and 61.1% (13.9% lateral tear, 44.4% medial tear, and 2.8% medial and lateral tear) subjects in the Clinifibre® and FiberWire® group, respectively (p=0.81). However, at the discretion of the principal investigator, the injured meniscus was repaired in 47.2% and 41.7% of subjects assigned to the Clinifibre® and FiberWire® group, respectively (p=0.64). Inside-out technique was mostly used for meniscal repair in both Clinifibre® (36.1%) and FiberWire® (27.8%) groups (p=0.30). Other techniques of meniscal repair, including meniscectomy, partial medial meniscectomy, or outside-in technique, were also performed. Other intraoperative and post-operative profile of the subjects is provided in Table 2.

**Table 2:** Intraoperative and post-operative profile of subjects Data are presented as mean±standard deviation or frequency (percentage). ACL, anterior cruciate ligament; MWU, Mann-Whitney U test

Subject profile	Clinifibre^®^ (n=36)	FiberWire^®^ (n=36)	Statistical test	P-value
Intraoperative				
ACL graft length (cm)	7.8±1.2	8.2±0.9	MWU	0.175
ACL graft diameter (cm)	0.8±0.1	0.8±0.1	MWU	0.502
Graft preparation time (minutes)	19.2±7.5	20.5±8.0	MWU	0.778
Graft type				
Single bundle hamstring	25 (69.4)	24 (66.7)	Chi-square	0.964
Double bundle hamstring	9 (25.0)	10 (27.8)		
Quadruple bundle hamstring	2 (5.6)	2 (5.6)		
Suture number for graft preparation	1.8±1.1	1.9±1.0	MWU	0.133
Length of surgery (hours)	1.9±0.6	1.7±0.5	MWU	0.141
Implants for femoral fixation				
Cortical button with adjustable loop	4 (11.1)	3 (8.3)	Chi-square	0.691
Cortical button with fixed loop	32 (88.9)	33 (91.7)		
Implants for tibial fixation				
Bioabsorbable screw	33 (91.7)	36 (100.0)	Chi-square	0.077
Metallic screw	3 (8.3)	0		
Post-operative				
Post-surgery onset of pain (hours)	2.4±1.5	2.6±2.1	t-test	0.977
Length of hospital stay (days)	2.9±1.5	3.1±1.5	MWU	0.669
Walk without crutches (days)	28.4±18.8	26.9±13.2	MWU	0.969
Number of stairs climbed at week 26	36.7±25.6 (n=34)	40.2±27.6	MWU	0.613
Return to normal activities (days)	23.8±18.4	24.6±17.5	MWU	0.520
Return to work (days)	70.9±53.4	74.3±51.8	MWU	0.735
Return to pre-injury sport (days)	206.7±96.4 (n=11)	186.8±113.7 (n=5)	t-test	0.511
Length of single leg-hop (cm) at week 52	59.6±35.4	66.8±44.1	MWU	0.058

**Figure 2:**
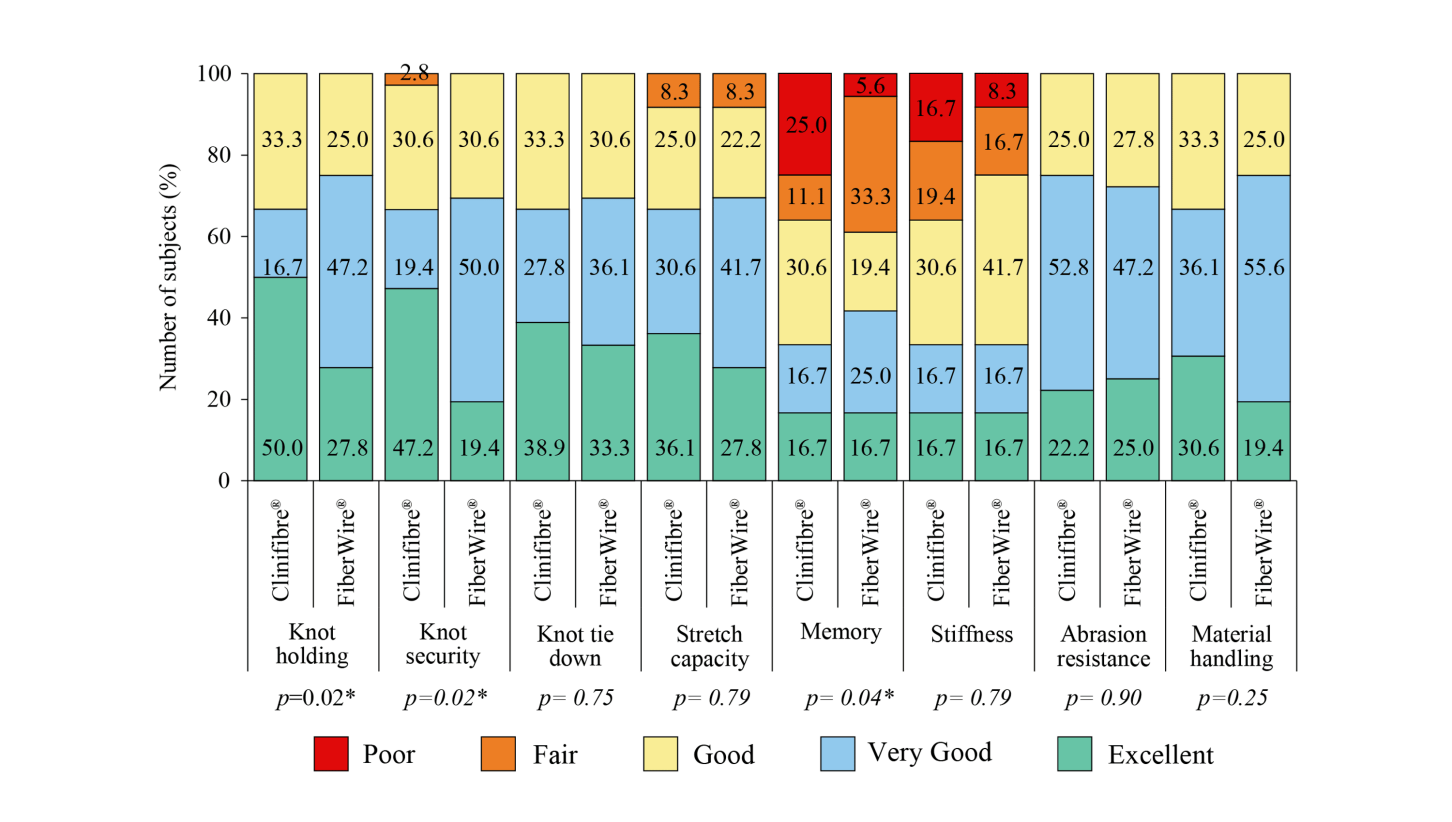
Intraoperative handling of Clinifibre® (n=36) and FiberWire® (n=36) sutures for graft preparation *p<0.05 The chi-square test was used

A higher proportion of excellent scores for intraoperative handling of Clinifibre® suture during graft preparation (knot holding, knot security, knot tie down, stretch capacity, and material handling) was found over FiberWire® suture. A greater proportion of very good scores for knot holding, knot security, knot tie down, stretch capacity, memory, and material handling of FiberWire® suture was noted in comparison to Clinifibre® suture (Figure 2).

Good outcome of surgery was recorded in both groups (p=1.00), with no record of requirement of blood transfusion, perioperative complications, and suture-related challenges.

**Figure 3:**
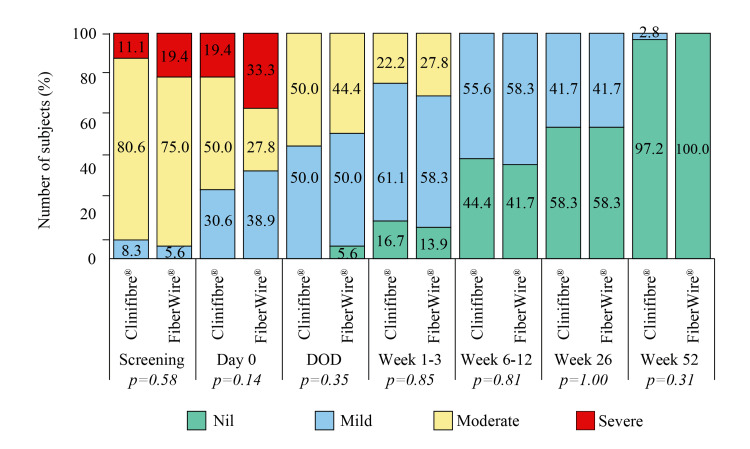
Grade of pain in the Clinifibre® (n=36) and FiberWire® (n=36) groups. The chi-square test was used DOD, day of discharge

No post-operative complications such as graft failure/rupture, unexpected poor range of movement/stiffness, excess bleeding, discoloration due to internal bleeding, and continued swelling were noted in this study. Slight swelling at week 26 in one subject in the Clinifibre® group and difficulty with knee movement due to slight pain in one subject in the FiberWire® group were recorded. All subjects had pain at the time of screening that decreased with each post-operative follow-up, and at the last follow-up, 97.2% and 100.0% of subjects in the Clinifibre® and FiberWire® groups had no pain (Figure 3). Mean pain VAS at screening (p=0.09) was 67.0±12.8 and 70.5±14.4 in the Clinifibre® and FiberWire® groups, respectively, which improved to 42.9±21.9 and 41.5±25.0, respectively, at DOD (p=0.95) and to 0.5±2.2 and 0.3±0.8, respectively, at week 52 (p=0.32). As a consequence, a reduction in the number of analgesics of 2.0±0.8 and 1.9±0.9 (p=0.53) on the day of surgery to 0.1±0.3 and 0.1±0.4 (p=0.57) on the last visit was noted in the Clinifibre® and FiberWire® groups, respectively. Grade of pain is presented in Figure 3.

**Figure 4:**
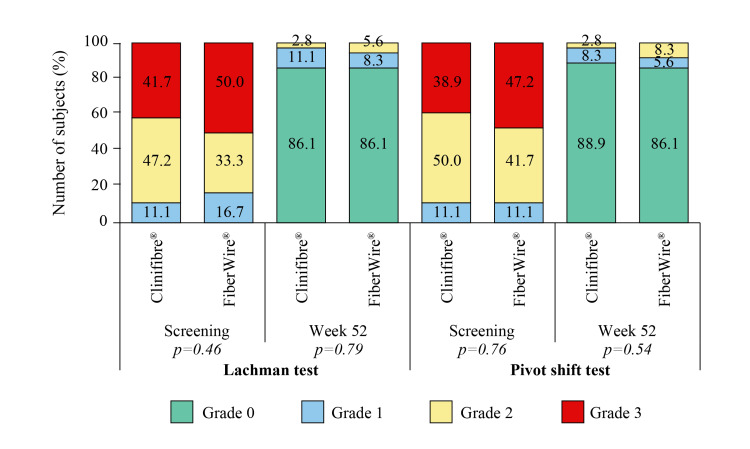
Lachman and pivot shift tests in the Clinifibre® (n=36) and FiberWire® (n=36) groups The chi-square test was used

Duration of hospital stay, as presented in Table 2, was comparable between the Clinifibre® and FiberWire® groups, with a minimum stay of one day and a maximum stay of six days in both groups; no readmission was required. The laxity of the operative knee has improved after ACLR, and at the last visit, the anterior translation of <3 mm (grade 0) was recorded in 86.1% of subjects in each study group. Similarly, poorer knee status at screening, measured by pivot shift test (grade 2 and 3), improved to grade 0 (normal) in the majority of subjects in both the Clinifibre® (88.9%) and FiberWire® (86.1%) groups at week 52 (Figure 4).

**Figure 5:**
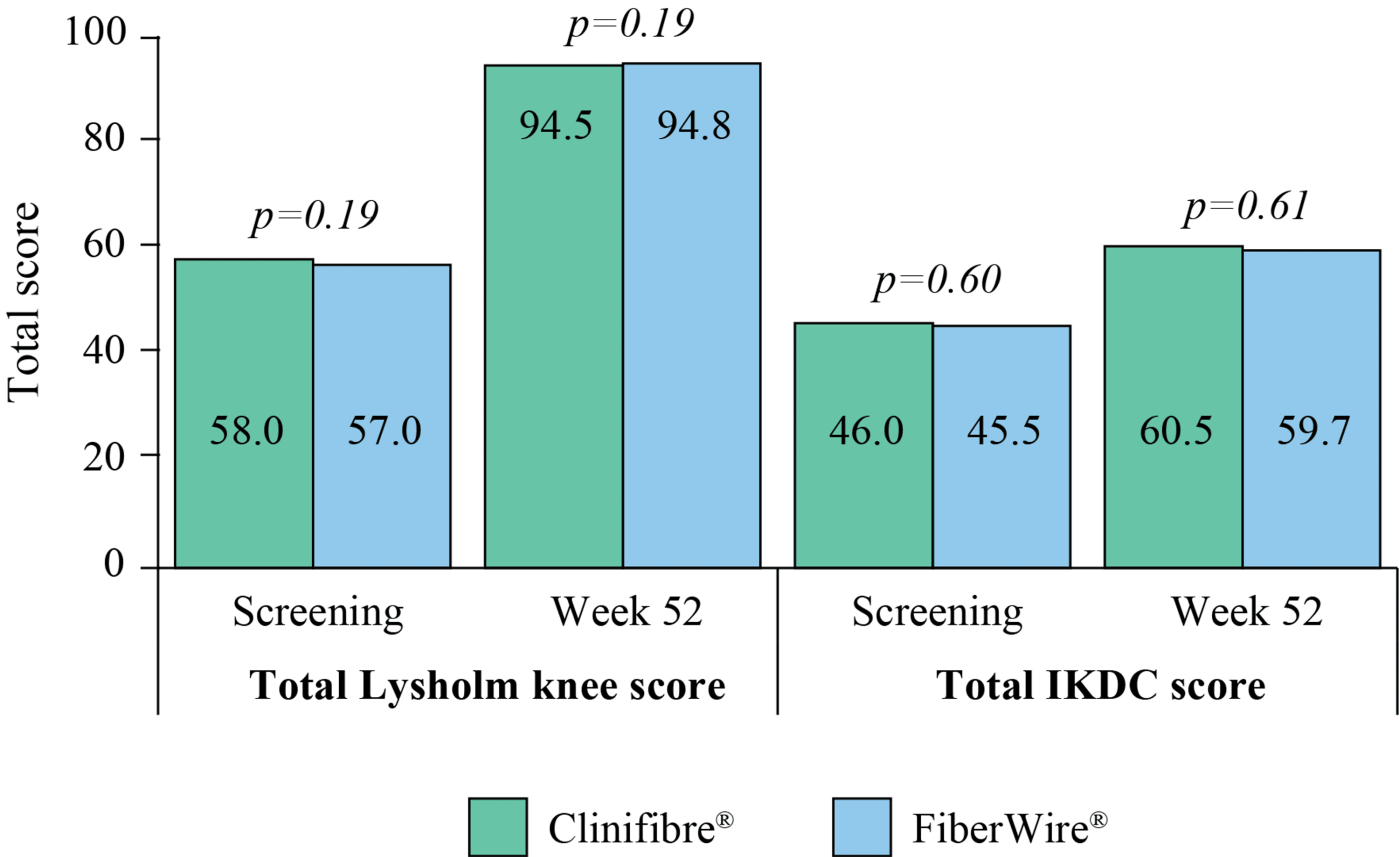
Total Lysholm and IKDC scores in the Clinifibre® (n=36) and FiberWire® (n=36) groups The t-test was used IKDC, International Knee Documentation Committee subjective knee evaluation form

The number of stairs climbed, as recorded at week 26, and the length of single leg-hop, as recorded at week 52, were comparable between the groups (Table 2). In the Clinifibre® and FiberWire® groups, mean Tegner activity level at screening was 2.1±1.0 and 2.0±1.1 (p=0.66), respectively, which improved to 5.3±1.5 and 5.1±1.1 (p=0.49), respectively, at week 52, indicating a better outcome with fewer symptoms or disability. In addition, total Lysholm knee and IKDC scores have shown improvement at post-ACLR week 52 in both the Clinifibre® and FiberWire® arms, as compared to the screening data (Figure 5).

**Figure 6:**
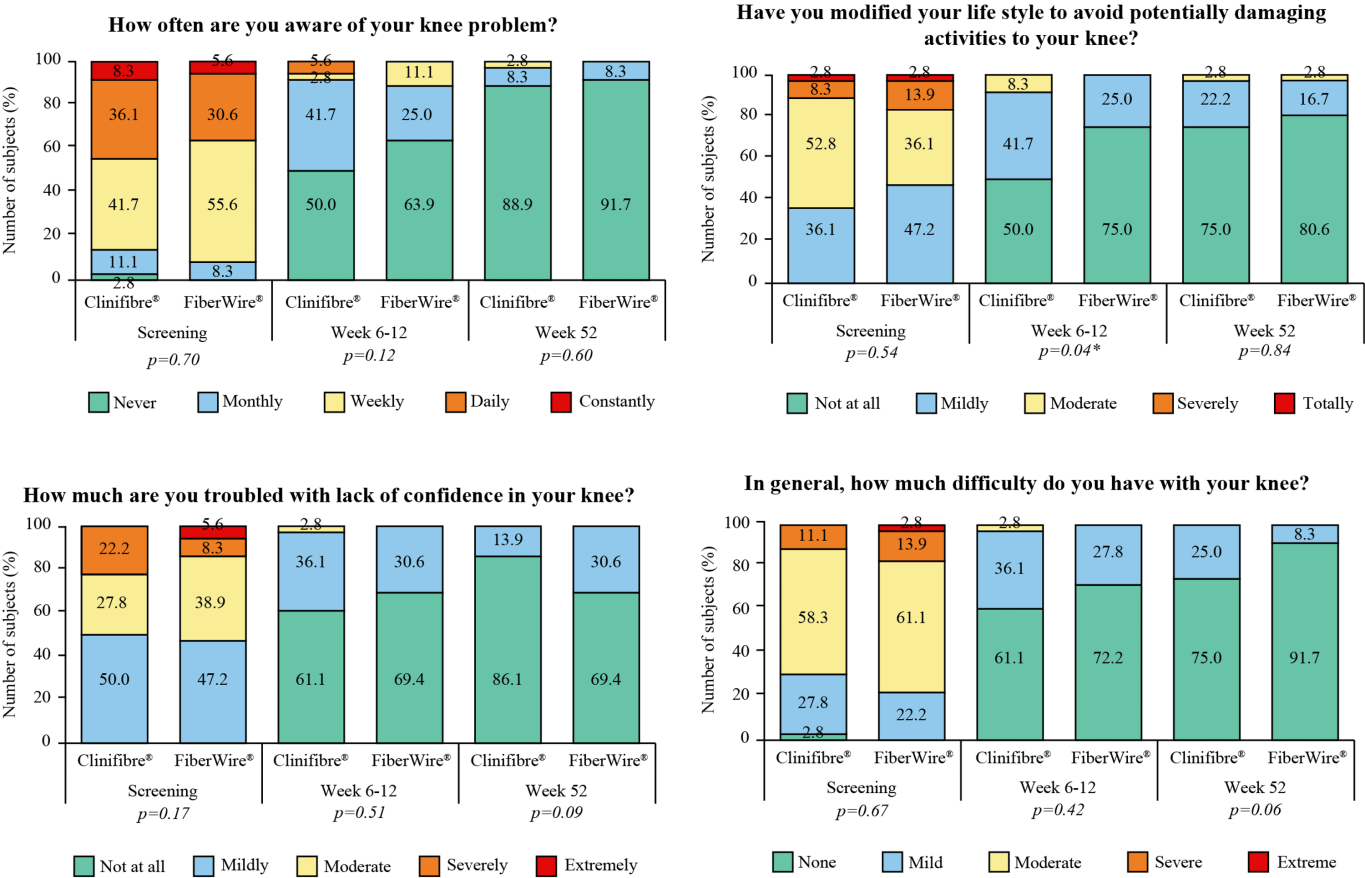
KOOS-quality of life in Clinifibre® (n=36) and FiberWire® (n=36) groups. *p<0.05. The chi-square test was used KOOS, Knee Osteoarthritis and Outcomes Score

Time to walk without crutches, return to normal day-to-day activities, return to work, and return to pre-injury sport were comparable between the study groups (Table 2). In comparison to screening data in both groups, a marked improvement in KOOS-QOL related to awareness of knee problem, lifestyle modification to avoid knee damage, lack of confidence in knee, and difficulty in knee was witnessed at week 52, as the majority answered “no” to the questions (Figure 6).

Safety

No incidence of serious or adverse device effect was recorded. The non-serious, device non-related AEs reported during the study span were as follows: fever in one subject in the Clinifibre® group and swelling in knee in one subject in each group.

## Discussion

ACL injury limits movement and physical activity levels, and results in pain, muscle weakness, changed biomechanics, and knee instability that place a great toll on the economy and patients’ health [20]. Since more than a decade, India has boasted of highly specialized centers, government academic institutes, private multispecialty/orthopedics hospitals, academic private sectors, and non-academic government sectors, focusing on ACLR surgeries [2]. According to a report from 2018, nearly 125,000 arthroscopic ACLR surgeries are performed in India every year [21]. With the increasing incidence of ACLR surgeries, the number of revision surgeries for a failed ACLR is also rising in India, owing to recurrent instability, graft failure, pain, and knee stiffness. Although infection rate after ACL repair is low, but not an insignificant cause for graft failure [22]. Rate of joint infection in ACL-reconstructed knee is 0.14% to 1.8%, but it has a major impact on ACLR, as the infection can damage cartilage and hinder ligamentization of the graft, resulting in graft failure [23]. Rate of infection was 2.8% in the present study (FiberWire® group), which occurred at the site of skin suturing. However, revision surgery was not essential to perform in the subject or any other study participants due to graft failure/rupture, stiffness, excess bleeding, or continued swelling. In addition, no AE in this study was deemed related to the intervention.

Biocompatibility and high wear resistance make UHMWPE sutures ideal for providing support to the medical implants [11]. In the opinion of the surgeons of the present study, both Clinifibre® and FiberWire® UHMWPE surgical sutures had similar properties of knot tie down, stretch capacity, stiffness, abrasion resistance, and material handling for graft preparation. However, the surgeons were comparatively more satisfied with knot holding, knot security, and memory of Clinifibre® suture for preparing the graft over FiberWire® suture. The suture augmentation technique is attributable to low pain, limited instability, and recovery time after ACLR surgery [24]. Hamstring tendon autografts that use only soft tissue section are associated with lower post-operative pain scores than other surgical techniques such as bone-patellar tendon-bone autografts, in which bone cut and extensive dissection in the anterior knee is used. Post-operative pain sometimes influences surgery outcome, viz. delay in returning to normal activities or work [25]. The pain reported after gaining consciousness post-surgery was diminished in the present study, along with less demand for using analgesics in both the Clinifibre® and FiberWire® groups. As a result, except one subject in the Clinifibre® group, who had mild pain at the last follow-up, none of the study participants had pain. Data of time to walk without crutches, return to normal day-to-day activities, return to work, return to pre-injury sport, and length of single hop test (at the end of the study) in both the Clinifibre® and FiberWire® groups further reflected the success of arthroscopic reconstruction in enhancing overall knee function of the subjects.

An ACLR is usually performed to reclaim anterior-posterior and rotational knee stability in order to return to pre-injury sports and other activities [6]. As the meniscus provides knee stability, the repair of concomitant medial or lateral meniscal tears during ACLR may aid in reducing the tibial translation [5]. A simple standardized test for assessing return to sports after ACL injury is the single leg-hop test [26]. Grade of laxity, negative pivot shift test, range of motion, strength of quadriceps, and time since surgery are the other key determinants of return to pre-injury sports following an ACLR [2]. A mean length of single leg-hop of around ≥60 cm was found after 52 weeks of ACLR in the present study. Evaluation of ACL status using the Lachman test typically suggested a torn ACL (anterior translation >2 mm) at screening in both Clinifibre® and FiberWire® groups. However, after ACLR, laxity of the operative knee improved, with a majority in both groups having anterior translation of <3 mm at the last visit. Pivot shift is preferred over the Lachman test for diagnosing the rotatory instability (axial and sagittal) and valgus stress [13]. An improvement from clunk (grade 2) and locked subluxation (grade 3) at the time of screening in most of the subjects to normal (grade 0) stability in majority at week 52 was also noted. The findings of time to return to daily activities, work, or pre-injury sports, and mobilization period with crutches further strengthen the positive role of arthroscopic ACLR in promoting functional outcomes of the subjects.

Lysholm knee score, IKDC, and KOOS-QOL scores indicate patient’s self-confidence in returning to pre-injury activities [1]. de Andrade et al. proposed isokinetic (extensor and flexor articular peak torque) correlation with Lysholm knee score. A significantly lower (36%) isokinetic deficit was reported in patients with a Lysholm score of >89 points after ACLR than of ≤89 points [27]. The mean Lysholm knee score was >94 (at Week 52) in both arms of the present study, indicating restored muscular strength post-ACLR. In terms of patient-reported outcomes of functional recovery and knee stability, Lysholm knee, Tegner activity, and IKDC scores showed no significant inter-group difference but marked improvement from screening to end of follow-up. The finding of KOOS-QOL has inferred that a substantial number of subjects experienced satisfactory recovery after ACLR, reflecting the success of graft preparation with UHMWPE sutures in arthroscopic reconstruction to enhance the overall knee function.

Besides the positive findings, it is important to acknowledge the study limitations. The sample size, though adequate for detecting differences in product performance of Clinifibre® and FiberWire®, may not be sufficiently powered to detect rare complications or long-term outcomes. Since this was a single-blind study, the knee surgeons were not blinded. There is a possibility that the surgeon might have favored intraoperative suture handling of one suture or another during graft preparation. The risk of SSI was low as this was a clean surgery and probably originated from contaminants or from skin colonists. Nevertheless, the comprehensive analyses of the study outcomes contribute to the evolving advancement in ACLR surgery, emphasizing the efficacy and safety of UHMWPE sutures for graft preparation. On the grounds of the present findings, generalized use of the Clinifibre® UHMWPE suture could be validated for a wider population and in all surgeries that use FiberWire® UHMWPE suture.

## Conclusions

Clinical equivalence of Clinifibre® and FiberWire® UHMWPE sutures is confirmed via the primary and secondary outcomes of this study. The observed improvements in laxity, pain, climbing stairs, knee stability, and QOL provided an insight into the overall recovery of subjects undergoing ACLR. The patient-reported functional outcomes further affirmed the efficacy of UHMWPE sutures for graft preparation during primary ACLR.
